# Evaluation of the EdgeSeq Precision Immuno-Oncology Panel for Gene Expression Profiling From Clinical Formalin-Fixed Paraffin-Embedded Tumor Specimens

**DOI:** 10.3389/fcell.2022.899353

**Published:** 2022-05-27

**Authors:** Yang Shi, Xiaopeng Ma, Wei Shen, Tengfei Liu, Liang Liang, Silu Liu, Zhirong Shen, Yun Zhang, Pei Zhang

**Affiliations:** BeiGene (Beijing) Co., Ltd., Beijing, China

**Keywords:** gene expression profile, tumor microenvironment, immunotherapy, platform evaluation, edgeseq PIP

## Abstract

Characterizing the tumor microenvironment (TME) of archived clinical tissues requires reliable gene expression profiling (GEP) of formalin-fixed paraffin-embedded (FFPE) samples. The EdgeSeq Precision Immuno-oncology Panel (PIP) is a targeted GEP assay designed for TME characterization but lacks widespread technical validation on a large cohort of clinical samples. Here, we evaluated its performance by exploring its concordance with multiple orthogonal platforms using 1,220 FFPE samples across various cancer types. Quantitative comparisons with RNA-seq and NanoString showed strong correlations at the sample level (median ρ = 0.73 and 0.81) and moderate correlations at the single-gene level (median ρ = 0.49 and 0.57). Gene signature analysis revealed high concordance with RNA-seq on widely used signatures for TME characterization and immune checkpoint inhibitor (ICI) efficacy prediction, though some genes in these signatures are not targeted by EdgeSeq PIP. From a histopathological viewpoint, the tumor/immune abundances derived from hematoxylin and eosin (H & E) staining were well recapitulated by the transcriptomic profiles assessed by EdgeSeq PIP. Furthermore, the mRNA level of PD-L1 assessed by EdgeSeq PIP was moderately correlated with the PD-L1 score (ρ = 0.65) estimated by immunohistochemistry (IHC); the mRNA level of CD8A aligned well (ρ = 0.55) with the IHC-derived abundance of CD8^+^ T cells. Overall, our results showed that EdgeSeq PIP generated well-correlated data with independent approaches at mRNA, protein, and histological levels, thus providing strong technical support for further using EdgeSeq PIP in biomarker studies and companion diagnostic (CDx) development.

## Introduction

Over the past years, immune checkpoint inhibitor (ICI)-based therapies have transformed the treatment landscape of cancer (Sanmamed and Chen, 2018). ICIs such as humanized monoclonal antibodies against cytotoxic T lymphocyte antigen 4 (CTLA4), programmed cell death protein 1 (PD-1) and programmed death ligand 1 (PD-L1) have demonstrated impressive efficacy and have been approved as first-line or second-line therapies for an ever-growing list of malignancies (Vaddepally et al., 2020). However, only a small fraction of patients benefits from ICI-based therapy, and there are urgent needs to identify the mechanisms driving response or resistance and develop new biomarkers to guide personalized therapy (Havel et al., 2019). Unraveling the landscape of immune cell subpopulations in the tumor microenvironment (TME) and investigating their interactions with tumor and stromal cells is a critical step in this process (Chen and Mellman, 2017).

TME characterization heavily relies on precise and comprehensive gene expression profiling (GEP). Among multiple GEP methods, RNA-seq has been established as the gold standard and is widely used in cancer research (Wang et al., 2009), such as The Cancer Genome Atlas (TCGA). However, in clinical practice, RNA-seq (and derived methods, such as RNA Exome) has several limitations. On the one hand, it requires large amounts of high-quality samples, which is usually not feasible in clinical trials in which most samples are formalin-fixed and paraffin-embedded (FFPE) and exhibit moderate-to-severe degradation (Evers et al., 2011). This requirement also contradicts the scarcity of tumor tissues in most clinical trials, especially when other typical biomarker assays, such as tumor mutation burden (TMB) and immunohistochemistry (IHC) assays are in competition (Aisner et al., 2016). On the other hand, RNA-seq is untargeted and thus not cost-effective for the development of companion diagnosis assay, as only a small proportion of genes are of strong interest (Thorsson et al., 2018).

Given these limitations of RNA-seq and the increasing demands for profiling immuno-oncology-related genes in clinical trials, targeted assays such as EdgeSeq PIP (HTG Molecular, Tucson, AZ), PanCancer IO 360 (NanoString, Seattle, WA) and Oncomine IRRA (Thermo Fisher Scientific, Waltham, MA), have emerged in recent years. These assays significantly reduced requirements for sample quality and quantity, thus mitigating the challenges of sample acquisition and making them clinically feasible. EdgeSeq PIP, which focuses on ∼1,300 key genes related to immuno-oncology, has the highest coverage among all these assays. By utilizing quantitative nuclease protection chemistry, EdgeSeq PIP quantifies RNAs *via* an extraction-free approach, which eliminates the risk of extraction bias induced by the removal of short or fragmented RNAs (Martel et al., 2002; Qi et al., 2016). The data generated from FFPE samples has good concordance with that from fresh-frozen samples. Moreover, the extraction-free approach circumvents the loss of RNA from sample during extraction; thus, less tissue input is required to generate an equivalent amount of RNA. Last, EdgeSeq PIP has the unique advantage of utilizing samples previously subjected to hematoxylin and eosin (H & E) or IHC staining, which further expands its clinical utility when samples are extremely limited (Qi et al., 2019). Although successfully used in several clinical trials (Wang et al., 2018; Desai et al., 2020; Garg et al., 2020; Martin-Broto et al., 2020; Song et al., 2021), the reliability of EdgeSeq PIP in real clinical settings and its concordance with other platforms have not been well studied. Previous studies either compared only EdgeSeq PIP with RNA-seq at the single-gene level or were limited by a small number of samples to draw solid conclusions (Anguiano et al., 2020; Ran et al., 2020). In this study, using 1,220 FFPE samples across several cancer types from clinical trials, we performed comprehensive comparisons of EdgeSeq PIP and multiple platforms and confirmed its fidelity at the RNA, protein, and histological levels.

## Materials and Methods

### Patient Cohort

Baseline FFPE samples were collected from the following seven clinical studies on tislelizumab monotherapy or tislelizumab combined with chemotherapy or anti-PD-L1 therapy: A317-001 (NCT02407990), A317-102 (NCT04068519), 900-101 (NCT03379259), A317-204 (NCT04004221), A317-205 (NCT03469557), A317-206 (NCT03432598), RATIONALE 307 (NCT03594747), and RATIONALE 309 (NCT03924986). The major cancer types in each study were summarized in Supplementary Table S1.

### Sample Preparation, Library Construction and Sequencing

All archived FFPE blocks were prepared as previously described. After confirming the presence of malignant cells by histological H & E staining, the samples were processed *via* standardized procedures for biomarker investigation. In general, a tumor content of at least 20% and sufficient tumor area (>20 mm^2^ for RNA-seq and >2.5 mm^2^ for EdgeSeq PIP) were required for sample inclusion.

For EdgeSeq PIP, sample processing, library construction and sequencing were performed in accordance with OP-00034, OP-00035, OP-00079 (HTG EdgeSeq instrument method). Briefly, tissues were scraped and lysed using lysis buffer from HTG Molecular Dianostics. Next, Proteinase K was added to digest proteins and remove potential contaminations. Gene-specific nuclease protection probes were then added to the lysed samples to form the probe-target RNA heteroduplexes, after which S1 nuclease was added to degrade non-hybridized molecules. Then samples were individually barcoded using a 16-cycle PCR, purified using Agencourt AMPure XP beads and loaded into Illumina MiSeq (Illumina, San Diego, CA) for 50 bp single-end sequencing.

For RNA-seq, RNA was extracted using the AllPrep DNA/RNA FFPE Kit (QIAGEN, Hilden, Germany). The amount of RNA was quantitated by the fluorescence method using Qubit RNA HS Assay Kit (Thermo Fisher Scientific, Waltham, MA), and the quality was assessed by Agilent 2,100 Bioanalyzer System (Agilent Technologies, Santa Clara, CA). Only samples with RNA >40 ng and DV200 >20% were included for downstream steps. rRNA depletion, cDNA synthesis and NGS library preparation were performed using the TruSeq RNA Exome (Illumina, San Diego, CA). The libraries were then loaded into HiSeq X Ten instrument (Illumina, San Diego, CA) for 150 bp paired-end sequencing.

For NanoString, all processing steps were performed according to the manufacturer’s instructions. In brief, RNA was extracted using RNeasy Mini Kit (QIAGEN, Hilden, Germany). The amount of RNA was quantitated by the fluorescence method using Qubit RNA HS Assay Kit (Thermo Fisher Scientific, Waltham, MA), and the quality was assessed by Agilent 2,100 Bioanalyzer System (Agilent Technologies, Santa Clara, CA). After hybridization with the PanCancer IO 360 Panel, sample analysis was performed on a nCounter Digital Analyzer (NanoString Technologies, Seattle, WA).

### EdgeSeq Precision Immuno-Oncology Panel Data Processing

Demultiplexed FASTQ files from the Illumina MiSeq were parsed by the EdgeSeq parser (HTG Molecular Diagnostics, Inc.). Three post-sequencing quality control metrics were derived and used for filtering samples: 1) >15% percentage of reads allocated to the positive process control probe; 2) >1.5 million reads; and 3) a relative standard deviation (RSD) of reads allocated to each probe >0.10. After removing samples not meeting these QC requirements, the raw read counts for each sample were transformed to the log2 counts per million (CPM) scale.

### RNA-Seq Data Processing

Adapters in raw reads were detected and trimmed by Trimmomatic (0.36) (Bolger et al., 2014). After trimming, reads shorter than 50 bp were removed. These reads were then mapped to the human genome (GRCh38) using STAR (2.7.10a) (Dobin et al., 2013). Gene expression was quantified using the RSEM workflow (1.3.3) with default parameters (Li and Dewey, 2011). Only samples with >1.5 million reads and >80% reads confidently mapped to the transcriptome were retained. Then, transcripts per million (TPM) values were log2-transformed for downstream analysis.

### Differential Expression and Gene Set Enrichment Analysis

Differentially expressed genes were identified following the limma-voom workflow (3.50) (Law et al., 2014; Ritchie et al., 2015). Gene set variation analysis (GSVA), single-sample gene set enrichment analysis (ssGSEA) and Z scores for each signature were calculated using the R package GSVA (1.42) by switching the “method” parameter (Hanzelmann et al., 2013). In addition to the signatures collected from specific studies, gene sets from the MSigDB-C2-Canonical Pathway (KEGG, BioCarta, PID, Reactome, WikiPathways) were also included in the signature analysis (Liberzon et al., 2011).

### Programmed Death Ligand 1 Immunohistochemistry

PD-L1 expression was assessed by the VENTANA PD-L1 (SP263) IHC assay (Ventana Medical Systems, Oro Valley, AZ, United States). The level of PD-L1 was then scored by the percentage of PD-L1 membranous staining on tumor cells (TC).

### CD8 Immunofluorescence

CD8 immunofluorescence analysis of FFPE samples was performed with CD8A antibody (SP57, Ventana 790-4460) in a College of American Pathologist (CAP)-controlled area within the Oncology and Immunology Unit of WuXi AppTec using the IF 6-colorWJJ-CD30 protocol on a Leica BOND Rx platform. Whole-slide images were acquired by Leica Aperio VERSA 8. Z1. Image analysis was performed using the HALO software package (Indica Labs, United States).

### Statistical Analysis

Correlation between two continuous variables was assessed by Spearman correlation. The interpretation of correlation was defined as: negligible (0–0.09), weak (0.10–0.39), moderate (0.4–0.69), strong (0.70–0.89) and very strong (0.90–1.00) (Schober et al., 2018). Differences in medians or continuous variables between two groups were assessed by non-parametric Wilcoxon rank-sum tests. The alpha level for all comparisons was 0.05 unless indicated otherwise. All statistical analyses and visualizations were performed with R (v.4.0.2).

## Results

### High Concordance Between EdgeSeq Precision Immuno-Oncology Panel and Other GEP Platforms at Gene Level

**FIGURE 1:**
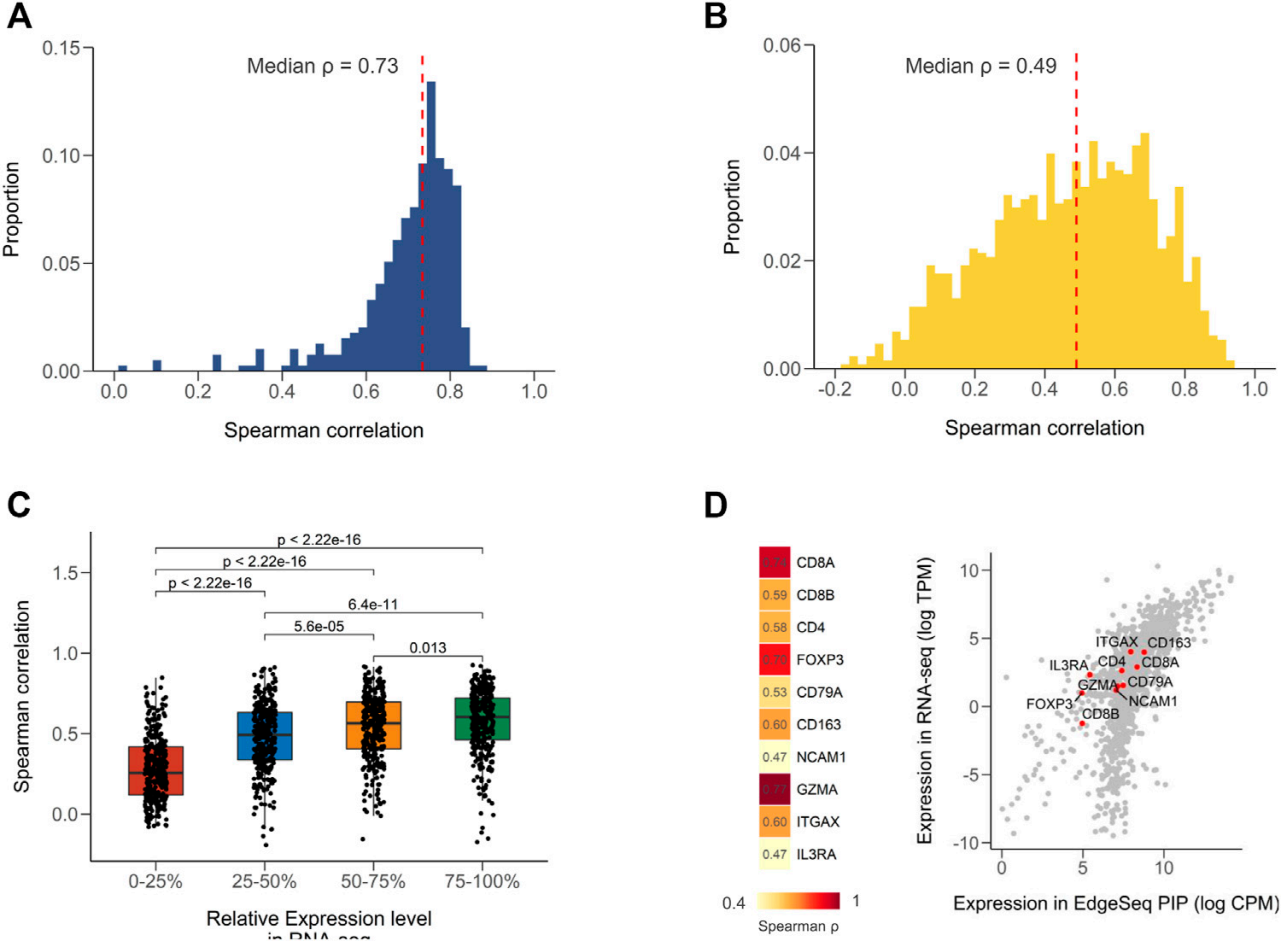
Concordance of EdgeSeq PIP with RNA-seq at gene level. **(A)** Distribution of sample-wise Spearman correlation coefficients between EdgeSeq PIP and RNA-seq. Dashed line represents the median value. **(B)** Distribution of gene-wise Spearman correlation coefficients between EdgeSeq PIP and RNA-seq. Dashed line represents the median value. **(C)** Boxplot showing the difference of Spearman correlation coefficients across genes stratified by their relative expression level (quartile) in RNA-seq. **(D)** Heatmap showing the Spearman correlation of key immune cell markers (left) and scatter plot showing their expression levels (right) in EdgeSeq PIP (*x*-axis) and RNA-seq (*y*-axis).

We first calculated Spearman correlation coefficients for all common samples between EdgeSeq PIP and RNA-seq (Supplementary Table S2). The strength of correlation was defined according to a widely used guiding rule for correlation interpretation in medical research (Materials and Methods) (Schober et al., 2018). Of the 395 samples, most had strong correlation (median ρ = 0.73) (Figure 1A). When assessing the concordance of the two platforms using a gene-by-gene approach, we found a moderate correlation (median ρ = 0.49) (Figure 1B and Supplementary Table S3), which was comparable with the result from a previous study comparing RNA-seq with NanoString (Kwong et al., 2018). We presumed that the weak correlation of some genes was partially attributed to their low expression levels. By stratifying genes according to their relative expression in RNA-seq, we found that the correlation coefficients significantly decreased as the expression level decreased (Figure 1C). The genes with expression levels in the lowest 25% only had a median correlation of 0.26 (Figure 1C). In addition, the dynamic range of gene expression measured by the median absolute deviation (MAD) also associated with the cross-platform correlation (Supplementary Figure S1A; Supplementary Table S3). This might indicate that correlation analysis was not suitable for numbers with low variances. After excluding genes with low expression (<1 TPM) and dynamic range (MAD <0.98, 25% quartile), the median correlation improved from 0.49 to 0.60 (Supplementary Figure S1B). Furthermore, considering the intended use of EdgeSeq PIP for profiling immune statuses in tumors, we evaluated the expression of several key immune markers and found all genes had moderate to strong correlations (ρ range from 0.47 to 0.77) (Figure 1D and Supplementary Figure S1C).

We also performed comparisons of EdgeSeq PIP and NanoString in another 17 samples and found strong correlations (median ρ = 0.81) using the sample-by-sample approach and moderate correlations (median ρ = 0.57) using the gene-by-gene approach (Supplementary Figures S2A,B, Supplementary Table S4). Similar to the results in RNA-seq, the immune markers assessed by EdgeSeq PIP also showed a high level of concordance with NanoString (6/9 genes had strong correlations with ρ > 0.7) (Supplementary Figure S2C). Taken together, our findings showed that EdgeSeq PIP had a high degree of agreement with RNA-seq and NanoString at the single-gene level.

### High Concordance Between EdgeSeq Precision Immuno-Oncology Panel and RNA-Seq for Characteristic Tumor Microenvironment Gene Signatures

**FIGURE 2:**
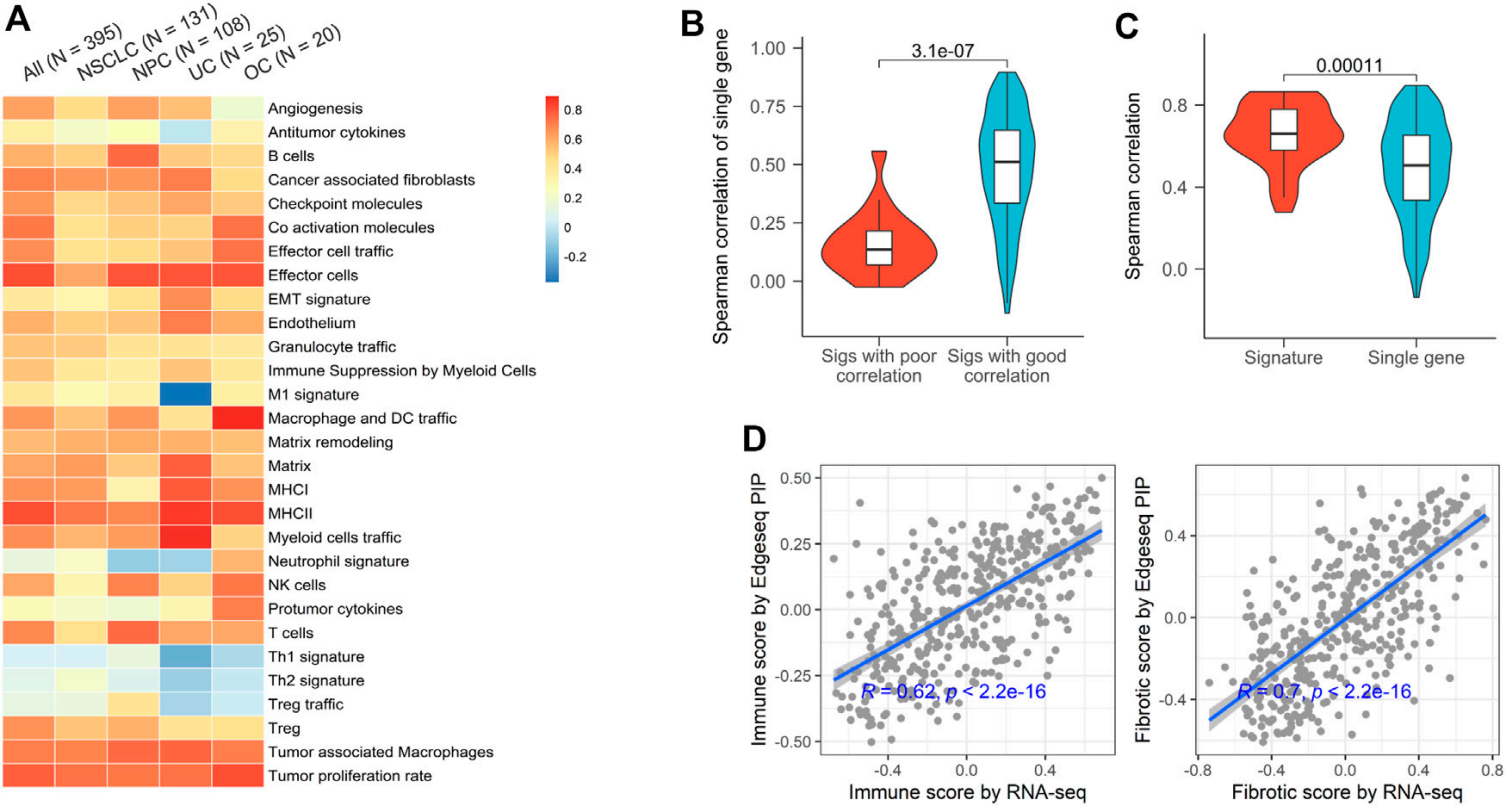
Concordance of EdgeSeq PIP and RNA-seq for TME characterizing signatures. **(A)** Heatmap showing the Spearman correlation coefficients of 29 TME-characterizing signatures between EdgeSeq PIP and RNA-seq. Columns represent the overall correlation and correlation in each major cancer type. **(B)** Violin plots showing the gene-wise Spearman correlation coefficients of genes in poor and well correlated signatures. **(C)** Violin plots showing the distribution of signature-derived and gene-derived Spearman correlation coefficients (between EdgeSeq PIP and RNA-seq). **(D)** Scatterplot showing the correlation of immune scores (left) and fibrotic scores (right) estimated by EdgeSeq PIP and RNA-seq data.

Gene signature, a set of genes with similar expression patterns or biological functions, have been widely used in TME profiling (Liberzon et al., 2011). Compared to single gene, signature-based approach has many significant advantages, including dimension reduction and greater biological interpretability. Here, we used 29 signatures that successfully classified the TMEs of tumors from TCGA and investigated the applicability of EdgeSeq PIP for TME subtyping (Bagaev et al., 2021) (Supplementary Table S5). First, we assessed the concordance of gene set variation analysis (GSVA) scores derived from EdgeSeq PIP and RNA-seq (Materials and Methods). Most signatures had moderate positive correlations (median ρ = 0.61), which persisted even after samples were divided into smaller groups according to cancer types (Figure 2A). Nevertheless, five signatures: neutrophil, Th1, Th2, Treg, and Treg traffic had negligible correlations (median ρ = 0.01) between the two platforms (Figure 2A). This result may have been due to the incomplete coverage of gene list in certain signatures by EdgeSeq PIP or to the involvement of genes with low correlations between the two platforms. We first explored whether the percentage of missed genes in each signature influenced the correlation. Surprisingly, the signatures with poor correlation did not have significantly higher rates of missing genes than the others (Supplementary Figure S3B). In contrast, most genes in these five signatures with worse correlations had lower RNA-seq expression levels and thus weaker correlations with EdgeSeq PIP at the single-gene level (Figure 2B and Supplementary Figure S3C). This result suggested that not the incompletion of genes in the signature but rather the correlation at single-gene level drove the concordance of signatures. After removing these weakly correlated signatures, the remaining signatures had significantly higher correlations as well as lower variations than individual genes (Figure 2C), which indicated that signature-based analysis had the property of noise reduction.

In the previous study, the TME of TCGA tumors were classified into 4 subgroups and annotated according to their enrichment of immune or fibroblast components (Bagaev et al., 2021). By averaging the scores of corresponding signatures (Supplementary Table S5), we derived “immune enrichment” and “fibroblast enrichment” score for each sample and found the scores generated by EdgeSeq PIP and RNA-seq correlated well (ρ = 0.62 and 0.70 separately) (Figure 2D). Taken together, the data from EdgeSeq PIP generated reliable signature scores and TME subtype results.

### High Concordance Between EdgeSeq Precision Immuno-Oncology Panel and Other Gene Expression Profiling Platforms Regarding Potential Immune Checkpoint Inhibitor-Predictive Gene Signatures

**FIGURE 3:**
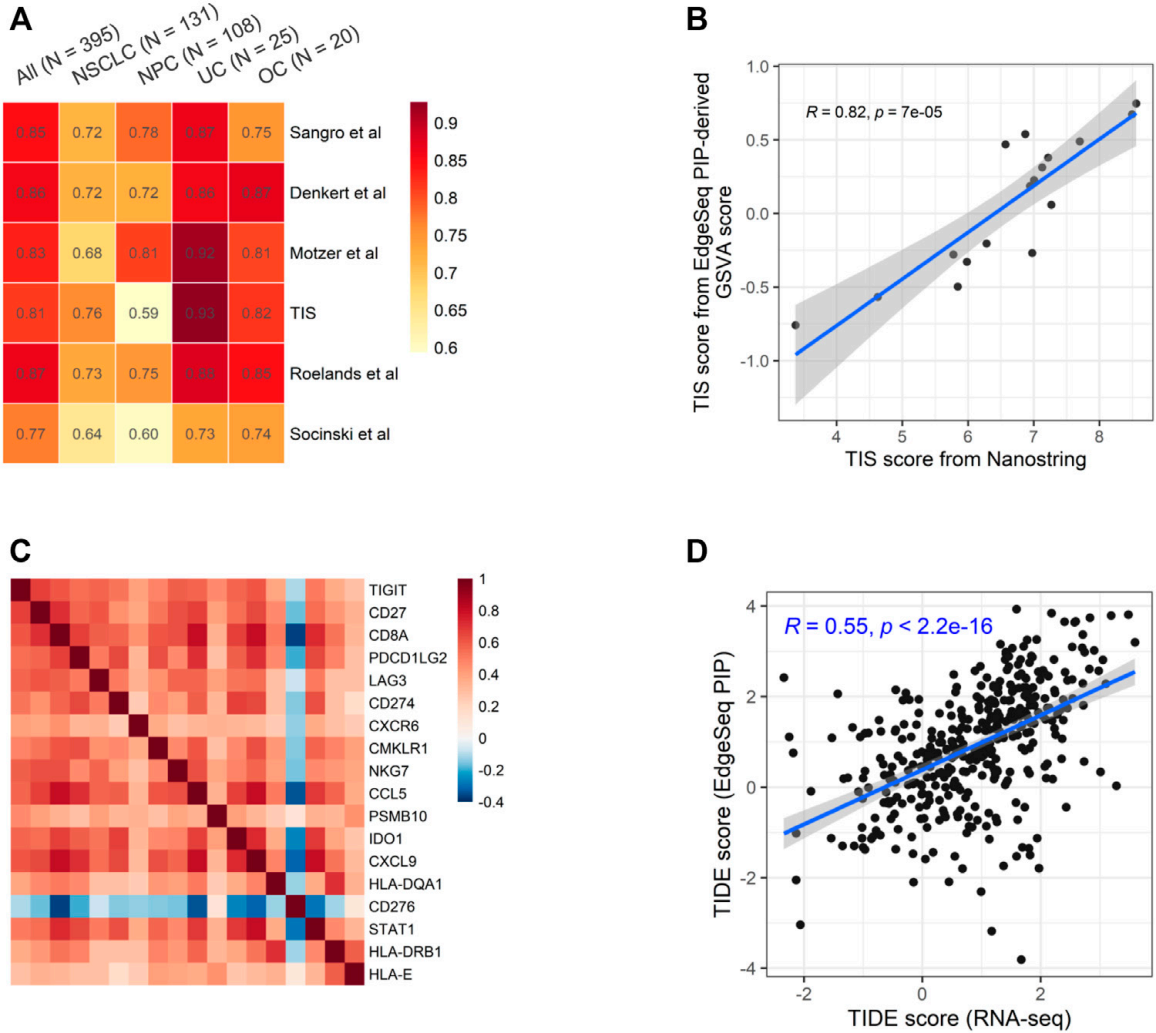
Concordance of EdgeSeq PIP and RNA-seq for potential ICI-predictive signatures **(A)** Heatmap showing the correlation coefficients of six ICI-predictive signatures between EdgeSeq and RNA-seq. Columns represent the overall correlation and correlation in each major cancer type. **(B)** Scatter plot showing the correlation of GSVA score derived from EdgeSeq PIP data with the official TIS scores from NanoString IO360 assay. **(C)** Heatmap showing the co-correlation of genes within TIS signature using RNA-seq data. **(D)** Scatter plot showing the correlation of TIDE score calculated from RNA-seq data and EdgeSeq PIP data.

We chose six well-known ICI-predictive signatures and studied the fidelity of calculating them using EdgeSeq PIP data (Supplementary Table S6) (Denkert et al., 2015; Cristescu et al., 2018; Socinski et al., 2018; Motzer et al., 2020; Roelands et al., 2020; Sangro et al., 2020). By calculating the correlation between signature scores derived from EdgeSeq PIP and RNA-seq, we found a high degree of concordance for all signatures (median ρ = 0.84) (Figure 3A and Supplementary Figure S4A). As expected, most genes in these signatures were well-correlated between the two platforms (median ρ = 0.57) (Supplementary Figure S4B).

Among these signatures, tumor inflammation signature (TIS) is originally derived from the NanoString platform using predefined algorithm and weight (Cristescu et al., 2018). Using the 17 samples with both EdgeSeq PIP and NanoString data, we applied GSVA on EdgeSeq PIP data to get “TIS-PIP score” and then compared it with the “TIS-NanoString score” derived from its original algorithm. We found a moderate correlation (ρ = 0.61) of TIS score despite of different platforms and algorithms (Figure 3B). In addition to GSVA, we also tested other methods for EdgeSeq PIP, such as ssGSEA and Z-score (Materials and Methods) and found consistent results (Supplementary Figures S5A,B). We reasoned that in addition to the cross-platform concordance at the single-gene level (Supplementary Figure S5C), the strong intra-signature correlation of genes could also account for the high correlation coefficient. Indeed, we found that except for *CD276*, all other 17 genes were moderately correlated with each other (median ρ = 0.46) (Figure 3C and Supplementary Figure S5D).

Additionally, we assessed tumor immune dysfunction and exclusion (TIDE), a computational framework integrating multiple signatures of tumor evasion that successfully predicts the responses of melanoma and lung cancer patients to ICIs (Jiang et al., 2018). We found that the TIDE scores generated by EdgeSeq PIP had moderate correlations with those generated by RNA-seq (ρ = 0.55) (Figure 3D). Overall, our results suggested that data from EdgeSeq PIP can be reliably used for most well-known ICI efficacy prediction algorithms.

### High Consistency Between EdgeSeq Precision Immuno-Oncology Panel and H & E Staining Results

**FIGURE 4:**
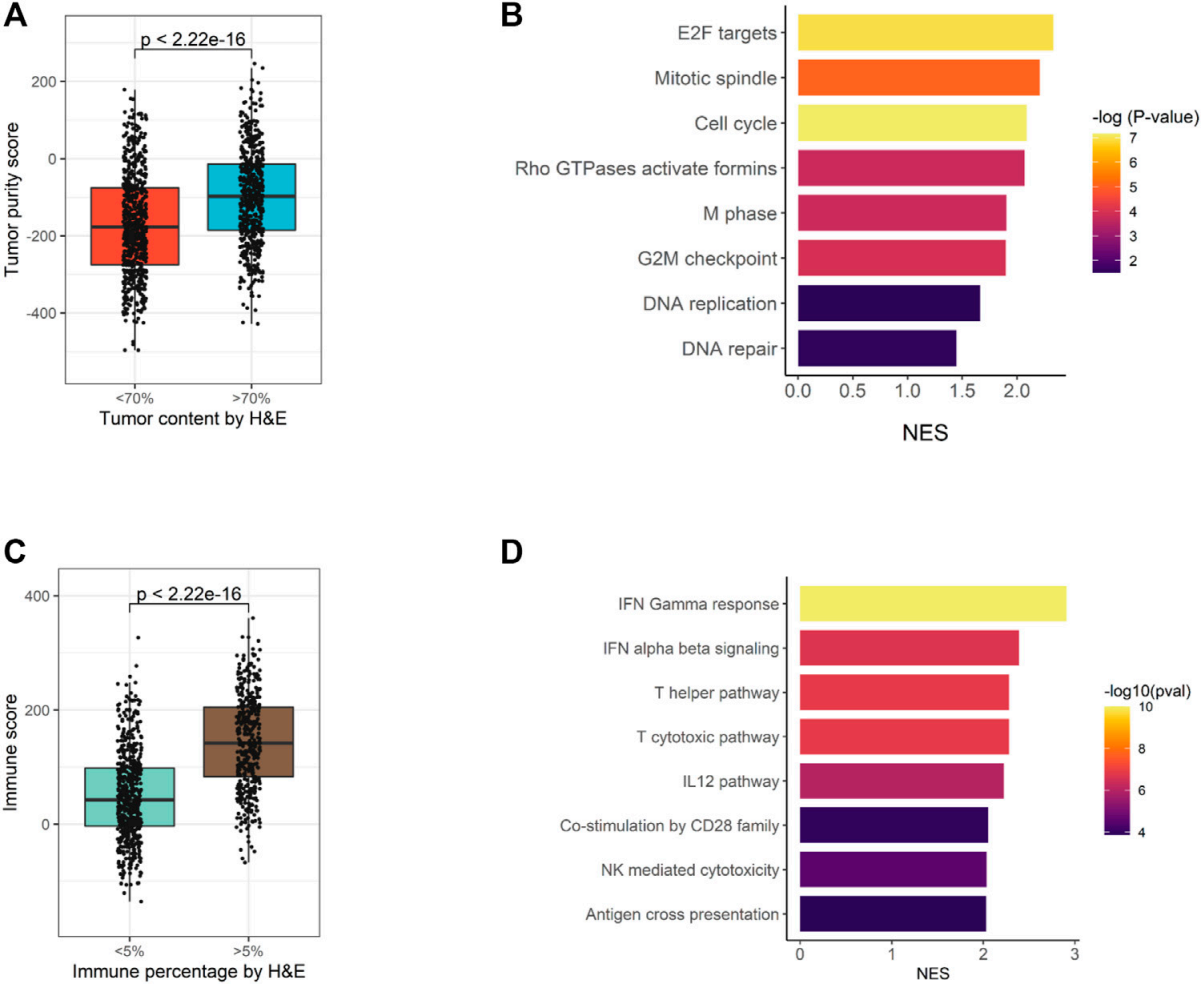
Consistency of EdgeSeq PIP data with H&E staining. **(A)** Boxplot showing the difference in tumor purity scores between samples with high (>70%) tumor percentage and low (<70%) tumor percentage. **(B)** Barplot showing the top eight pathways enriched in tumors with high (>70%) tumor percentage. **(C)** Boxplot showing the difference in immune scores between samples with high (>5%) immune percentage and low (<5%) immune percentage. **(D)** Barplot showing the top eight pathways enriched in tumors with high (>5%) immune percentage.

For 1,174 samples assessed by EdgeSeq PIP, H & E staining was also performed to evaluate the percentages of tumor cells and immune cells. Here, we assessed whether the H&E staining results could be recapitulated by EdgeSeq PIP at mRNA level. First, we used ESTIMATE (Yoshihara et al., 2013) to calculate the tumor purity score for each sample based on EdgeSeq PIP data. We found consistent result with H & E staining: the samples with a high tumor percentage (>70%, assessed by H & E) had significant higher tumor purity scores than those with low tumor percentage (<70%) (Figure 4A). In addition, we identified the differentially expressed genes between these two groups and performed gene set enrichment analysis (GSEA) (Materials and Methods) (Supplementary Table S7). Our results showed that cell cycle-related pathways were enriched among the genes upregulated in samples with >70% tumor percentage (Figure 4B and Supplementary Figures S6A,B). This finding aligned with the H & E result as most tumor cells had an extremely high proliferation rate.

Similarly, we studied the concordance of EdgeSeq PIP with the percentage of infiltrated immune cells estimated by H & E. On the one hand, tumors with >5% immune cell percentage (by H & E) had significant higher ImmunoScores (calculated by ESTIMATE) than others (Figure 4C). On the other hand, immune-related pathways and representative immune marker genes such as *CD8A*, *STAT1* and *GZMK* were up-regulated in tumors with >5% immune percentage (Supplementary Figures S6C,D, Supplementary Table S8). Overall, this evidence suggested that the abundance of tumor or immune cells assessed by histological methods was well reflected by EdgeSeq PIP.

### High Consistency Between EdgeSeq Precision Immuno-Oncology Panel and Immunohistochemistry

**FIGURE 5:**
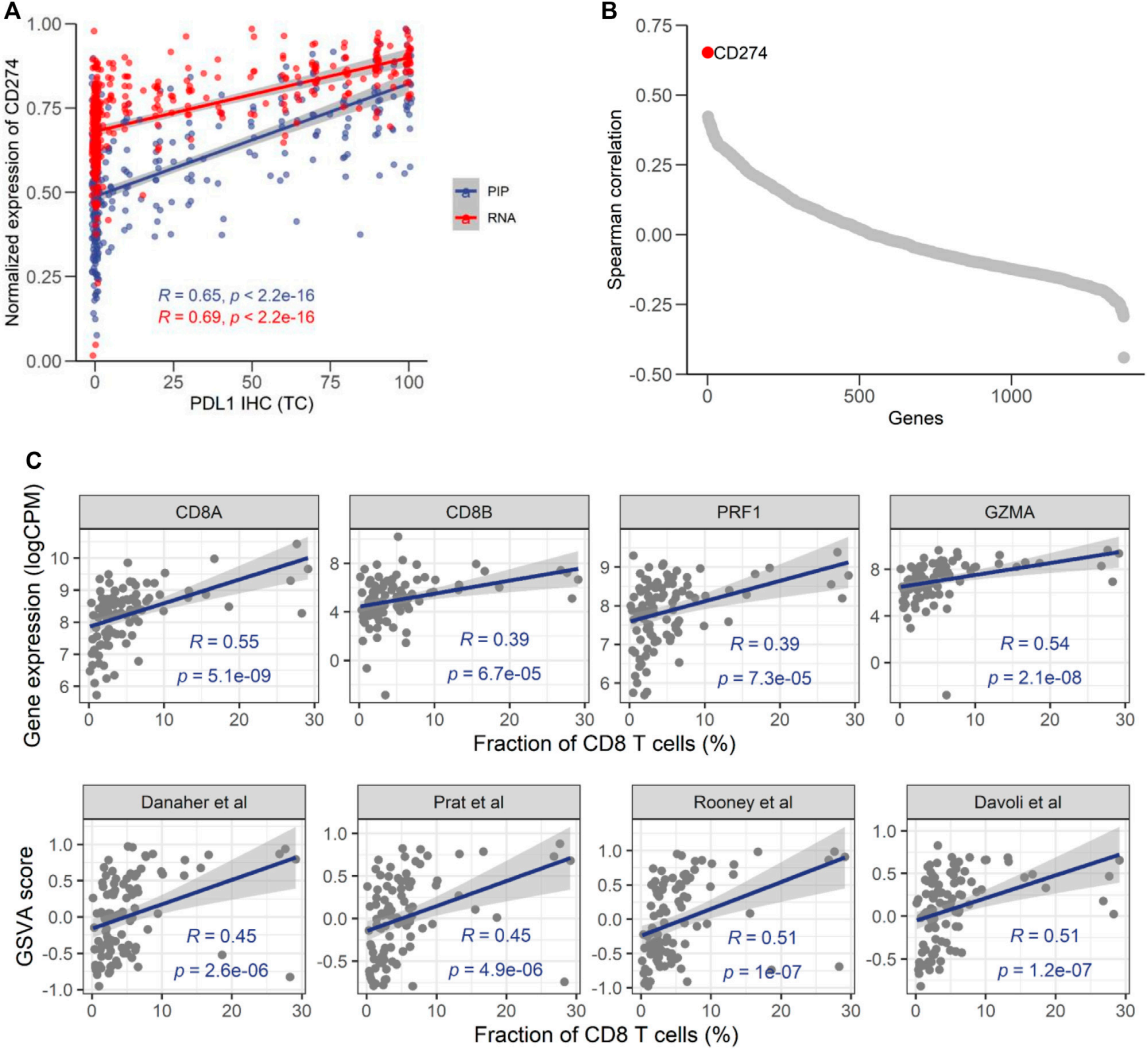
Consistency of EdgeSeq PIP with PD-L1 IHC and CD8 IHC. **(A)** Correlation of the CD274 mRNA expression level from EdgeSeq PIP (blue) and RNA-seq (red) with the PD-L1 TC score. **(B)** Spearman correlation coefficients (*y*-axis) of mRNA expression level assessed by EdgeSeq PIP with the PD-L1 level assessed by IHC. Each dot represents a gene and genes were ordered according to their Spearman correlation coefficients (*x*-axis). **(C)** Scatterplot showing the correlation of CD8 related genes (top) and signatures (bottom) with CD8^+^ T cell fraction estimated by IHC (%).

PD-L1 IHC score has been widely used for predicting responses to ICI-based therapies. A previous study showed that the mRNA level of PD-L1 assessed RNA-seq had good concordance with its protein level assessed by IHC (Conroy et al., 2019). Therefore, the correlation with PD-L1 IHC could be used to evaluate the fidelity of NGS-based approaches. In 369 samples with PD-L1 IHC data (Supplementary Figure S8), we found that the percentage of PD-L1 positive tumor cells had a moderate correlation (ρ = 0.65) with the mRNA level of PD-L1 (*CD274*) assessed by EdgeSeq PIP (Figure 5A), which was comparable to its association with RNA-seq (ρ = 0.69). It suggested that the remaining lack of correlation was mainly due to the discrepancy between mRNA and protein, rather than technical issues specific to platform. In addition, we calculated the correlation of PD-L1 IHC with every gene and found that *CD274* had the highest correlation coefficient among all genes (Figure 5B), which further suggested the fidelity of EdgeSeq PIP data.

To further investigate the concordance of EdgeSeq PIP with IHC, we assessed another important immuno-oncology marker, the fraction of CD8^+^ T cells, by CD8A IHC in another 98 tumors (Supplementary Figure S9). We investigated the correlation of CD8 abundance estimated by IHC with the expression level of 4 traditional CD8^+^ T cell markers (*CD8A*, *CD8B*, *PRF1*, *GZMA*) from EdgeSeq PIP and the EdgeSeq PIP-derived GSVA scores of 4 well-known CD8^+^ T signatures (Supplementary Table S9) (Rooney et al., 2015; Danaher et al., 2017; Davoli et al., 2017; Prat et al., 2017). All estimates for CD8^+^ T cells had significant positive correlations with the IHC results (Figure 5C). Interestingly, *CD8A* outperformed the other genes (ρ = 0.55) and even the gene signatures. This result suggested that at least for data generated by EdgeSeq PIP, the expression level of *CD8A* is sufficient for estimating the fraction of CD8^+^ T cells in tumors. Overall, our results indicated that the data generated by EdgeSeq PIP were comparable to IHC data and could be used as a complementary method for evaluating the protein levels of PD-L1 and the CD8^+^ T cell fraction.

## Discussion

Gene expression profiling plays an important role in immuno-oncology related biomarker studies, as it helps to elucidate the TME landscape, and many predictive biomarkers have been derived for ICI-based therapies. As clinical specimens in certain cancers are obtained from core needle biopsies of small size and suboptimal quality, tissue availability restricts the clinical application of traditional GEP methods (e.g., RNA-seq). EdgeSeq PIP, an assay focused on well-studied genes related to immuno-oncology, uses an extraction-free technology to minimize sample requirements. This method mitigates the challenge for sample acquisition and has been widely used in clinical trials.

In this study, we provided evidence that EdgeSeq PIP is a robust assay with a high degree of concordance with multiple platforms, including RNA-seq, tumor/immune cell fraction estimation by H & E staining, PD-L1 scoring and CD8^+^ T cell abundance estimation by IHC. By analyzing data from 395 clinical FFPE samples, we found that EdgeSeq PIP showed good correlation with RNA-seq at the single-gene level, especially after excluding genes with low expression levels or dynamic ranges. Importantly, we demonstrated that EdgeSeq PIP, with incomplete coverage of genes in some signatures, still generated signature scores that were well correlated with those of RNA-seq. This result indicated that EdgeSeq PIP data can be reliably utilized for signature-based analyses of TME subtypes and ICI efficacy prediction. In addition, we found that EdgeSeq PIP aligned well with the overall percentage of tumor/immune cells determined by H & E staining. Furthermore, our results showed that the RNA levels determined by EdgeSeq PIP aligned well with the protein levels determined by the IHC assessment of two important immunotherapy biomarkers, the fraction of PD-L1-positive tumor cells and CD8^+^ T cells.

As the method of choice for transcriptome probing, RNA-seq has always been used as a reference standard to benchmark other GEP assays. In our results, EdgeSeq PIP correlated well with RNA-seq except for a small proportion of genes with low expression levels. This result was within expectations, as true signals for genes with low expression are hard to distinguish from noise, which would diminish the correlation (Kwong et al., 2018). In addition, loss of degraded transcripts during RNA extraction, especially for low-expressing genes in archived FFPE samples, could further exacerbate the discordancy between the two platforms. There was evidence that EdgeSeq PIP, using its extraction-free technique, offer superior sensitivity on these “discordant” genes (Ran et al., 2020). Nevertheless, such genes should be used with caution and should even excluded from some analyses involving data from both RNA-seq and EdgeSeq PIP.

In this study, we found that the gene signature score derived from EdgeSeq PIP was strongly correlated with that from RNA-seq, although some genes in given signatures were not covered by EdgeSeq PIP probes. We reasoned that such result was attributed to the coexpression of genes within the signature (e.g., TIS). Such coexpression led to gene “redundancy” and made the signature score calculation robustly susceptible to gene dropout and different algorithms. Though some poorly correlated genes resulted in a low correlation among certain signatures, after their exclusion, most signatures defined based on RNA-seq data could be directly transferred and used on EdgeSeq PIP data. However, we must admit that the signatures used in our study were limited to those related to immuno-oncology, which predominantly consisted of surface markers sharing similar expression patterns. For other signatures, such as cellular signaling pathways, the effect of “gene dropout” needs to be further investigated.

PD-L1 IHC has been used to predict responses to ICI-based therapy. A previous study revealed that the expression of PD-L1 measured by RNA-seq had a strong association with that measured by IHC (Conroy et al., 2019). Here, we found that the mRNA level of PD-L1 generated by EdgeSeq PIP had a high correlation with the PD-L1 IHC level as well. Although a biological gap exists between mRNAs and proteins, EdgeSeq PIP at least provides another layer of PD-L1 quantification that can be exploited for the prediction of ICI efficacy. In addition, EdgeSeq PIP quantifies PD-L1 transcripts without the need for subjective scoring and cell type discrimination by pathologists, which might introduce substantial discrepancy across studies (Hirsch et al., 2017).

In summary, for the first time, we comprehensively benchmarked EdgeSeq PIP with multiple platforms using large-scale clinical FFPE samples, and the results are reflective of its actual performance in clinical practice. Our results showed that EdgeSeq PIP generated data comparable to those generated by classical methods. Considering its low sample requirement and cost efficiency, the technology would get an ever-increasing application for biomarker studies in clinical trials.

## Data Availability

The original contributions presented in the study are included in the Supplementary Material, further inquiries can be directed to the corresponding authors.
