# Supplementary material for: Evaluation of the EdgeSeq Precision Immuno-Oncology Panel for Gene Expression Profiling From Clinical Formalin-Fixed Paraffin-Embedded Tumor Specimens
Source: Front Cell Dev Biol. 2022 May 27;10:899353. doi: 10.3389/fcell.2022.899353 (PMC9197216; doi:10.3389/fcell.2022.899353)
Supplement: Supplementary file 2 [file DataSheet1.docx]

Supplementary Material

# Supplementary Tables

***Tables were included in an additional Excel file.***

**Supplemental Table S1.** **The number of patients stratified by cancer type in each trial.** The value in each cell represents the patient number, Rows are trials and columns are cancer types.

**Supplemental Table S2.** **The intersection of samples assessed by different assays.** The value in each cell represents the number of patients assessed simultaneously by the two assays in corresponding row and column.

**Supplemental Table S3.** **The statistics of gene correlation between EdgeSeq PIP and RNA-seq.** Median expression, median absolute deviation (MAD) and Spearman correlation coefficients of each gene were listed.

**Supplemental Table S4.** **The statistics of gene correlation between EdgeSeq PIP and NanoString.** Median expression, median absolute deviation (MAD) and Spearman correlation coefficients of each gene were listed.

**Supplemental Table S5. Information of the 29 signatures used for TME subtyping.** The genes in each signature were listed in the second column. The classification of “immune” or “fibrotic” signatures were listed in the third column.

**Supplemental Table S6. Information of the 6 ICI predictive signatures.** The genes in each signature were listed in the second column.

**Supplemental Table S7. Differentially expressed genes between samples with high/low tumor percentage.** The log2 fold changes (high vs low) and P values of each gene were listed.

**Supplemental Table S8. Differentially expressed genes between samples with high/low immune percentage.** The log2 fold changes (high vs low) and P values of each gene were listed.

**Supplemental Table S9. Information of the 4 CD8^+^T cell signatures.** The genes in each signature were listed in the second column.

# Supplementary Figures


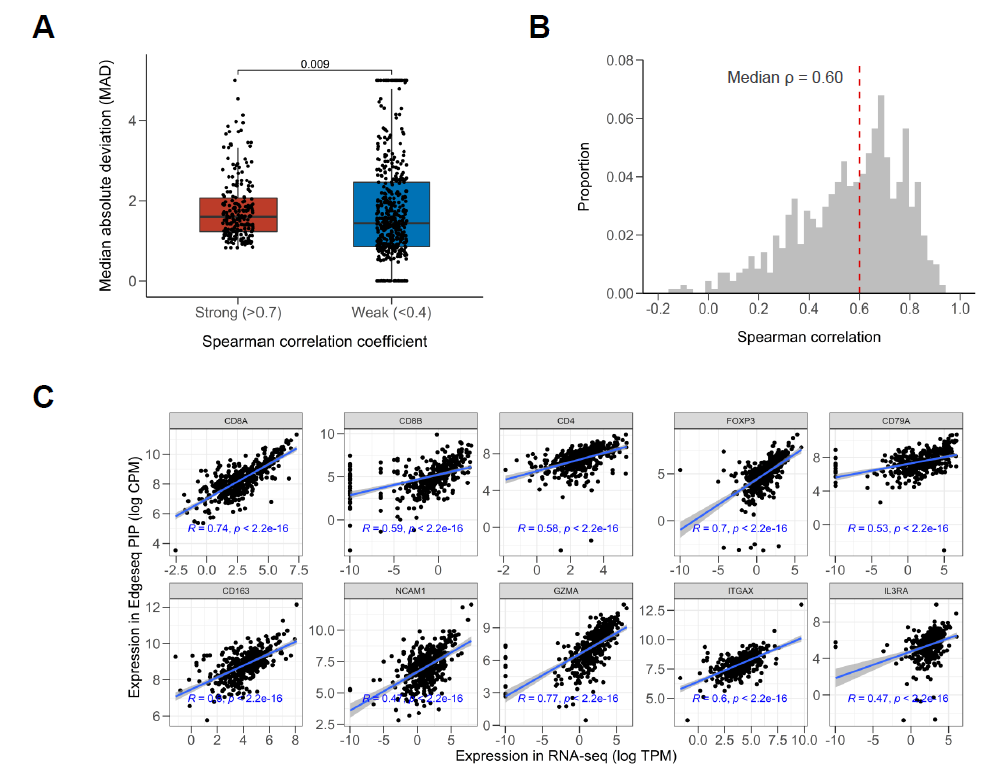


**Supplemental Figure S1. Concordance of EdgeSeq PIP with RNA-seq at gene level. (A)** Boxplot showing the difference in median absolute deviation (MAD) between genes with high correlation coefficients and those with low correlation coefficients. **(B)** Distribution of gene-wise Spearman correlation coefficients between EdgeSeq PIP and RNA-seq after excluding genes with < 1 FPKM and <1.04 MAD. **(C)** Scatterplot showing the correlation of 10 immune markers between EdgeSeq PIP and RNA-seq


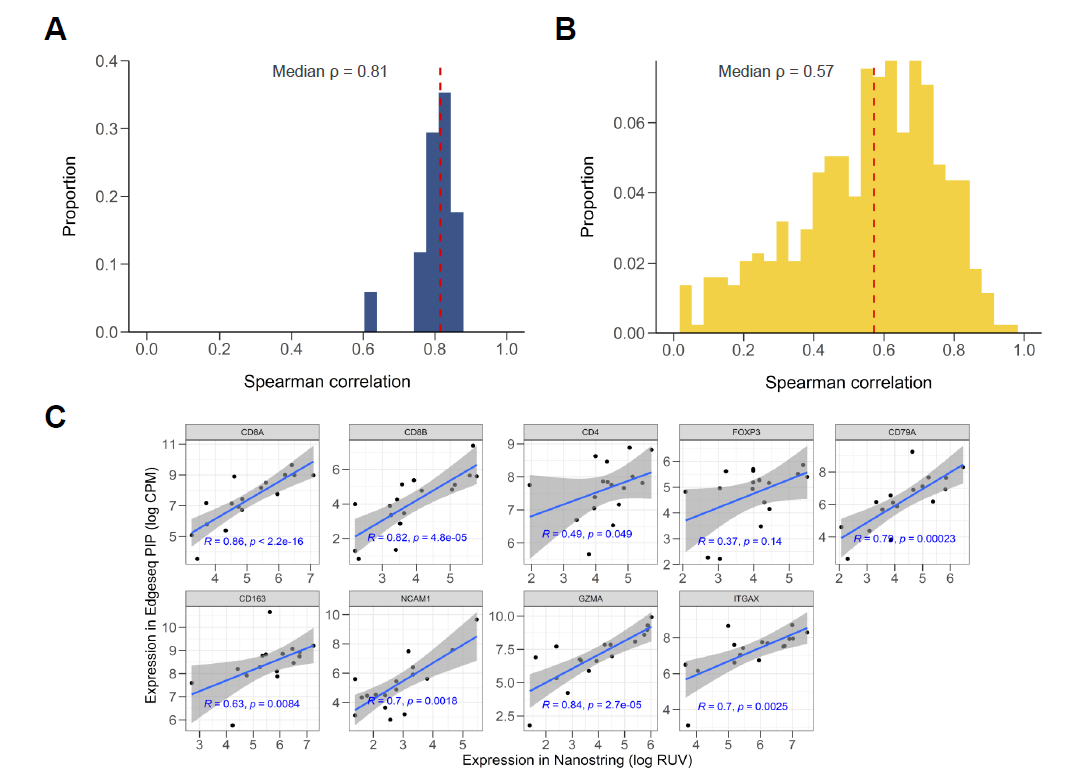


**Supplemental Figure S2. Concordance of EdgeSeq PIP with NanoString at gene level (A)** Distribution of sample-wise Spearman correlation coefficients between EdgeSeq PIP and Nanostring. Dashed line represents the median. **(B)** Distribution of gene-wise Spearman correlation coefficients between EdgeSeq PIP and NanoString Dashed line represents the median. **(C)** Scatterplot showing the correlation of 9 immune markers between EdgeSeq PIP and NanoString.


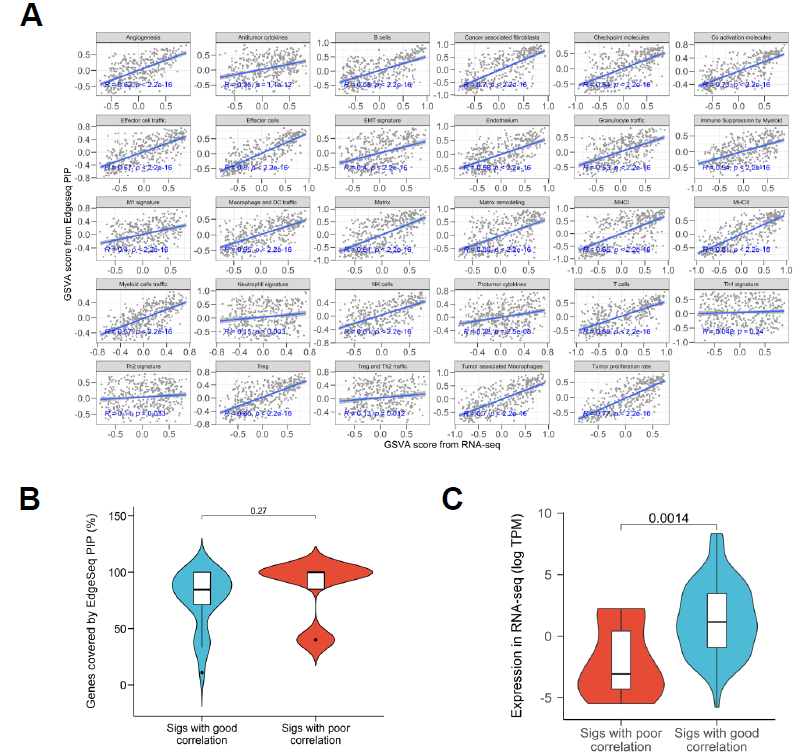


**Supplemental Figure S3. Concordance of EdgeSeq PIP and RNA-seq for TME-characterizing signatures**. (A) Scatterplot showing the correlation of 29 TME-related signatures between EdgeSeq PIP and RNA-seq. (B) Violin plot showing the percentage of genes covered by EdgeSeq PIP between poor and well correlated signatures. (C) Violin plot showing the expression level of genes in signatures with poor correlation and good correlation.


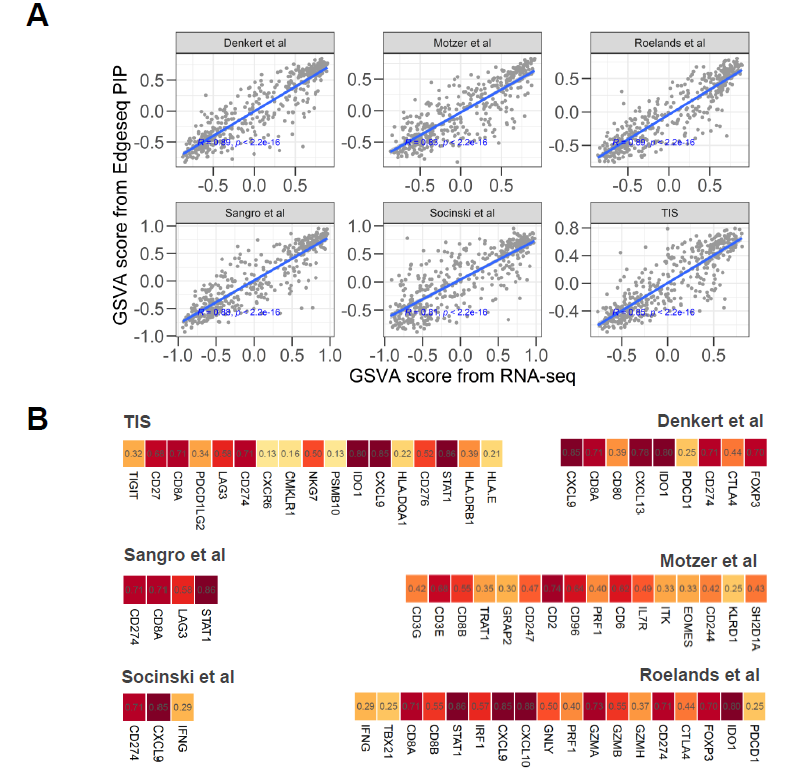


**Supplemental Figure S4. Concordance of EdgeSeq PIP and RNA-seq for potential ICI-predictive signatures. (A)** Scatterplot showing the correlation of 6 ICI-predictive signatures between EdgeSeq PIP and RNA-seq. **(B)** Heatmap showing the Spearman correlation coefficients of genes in each ICI-predictive signature between EdgeSeq PIP and RNA-seq.


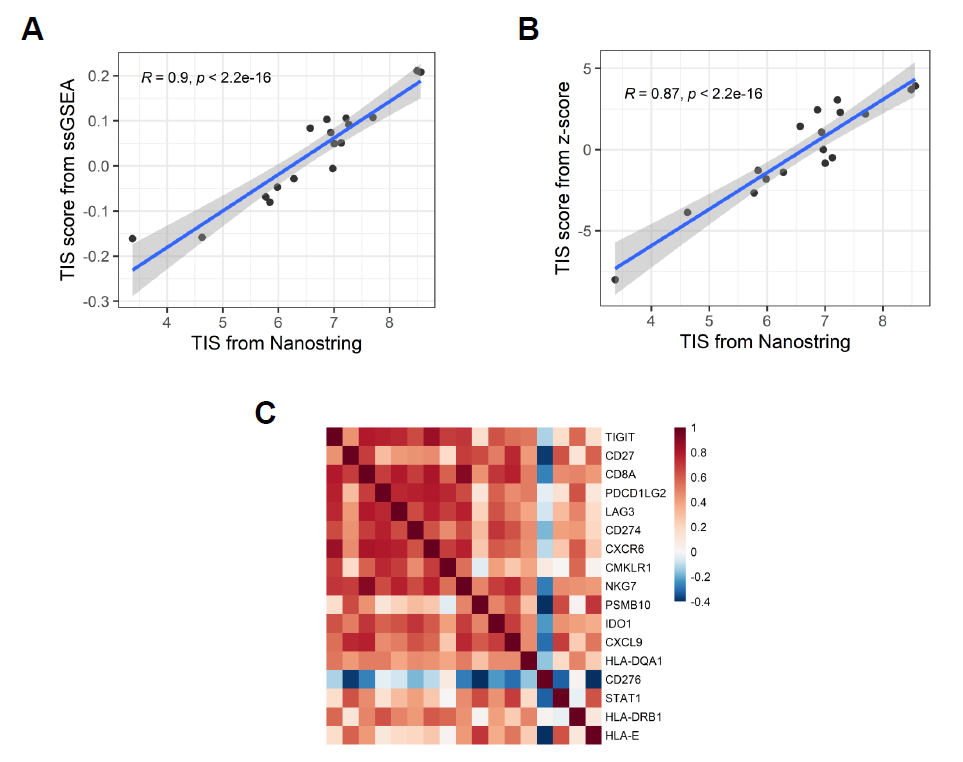


**Supplemental Figure S5. Concordance of EdgeSeq PIP and NanoString for TIS signature (A)** Scatterplot showing the correlation of TIS signature between ssGSEA score derived from EdgeSeq PIP and the official score derived from NanoString IO360 algorithm. **(B)** Scatterplot showing the correlation of TIS signature between Z-score derived from EdgeSeq PIP and the official score derived from NanoString IO360 algorithm. **(C)** Heatmap showing the co-correlation of genes within TIS signature using EdgeSeq PIP data.


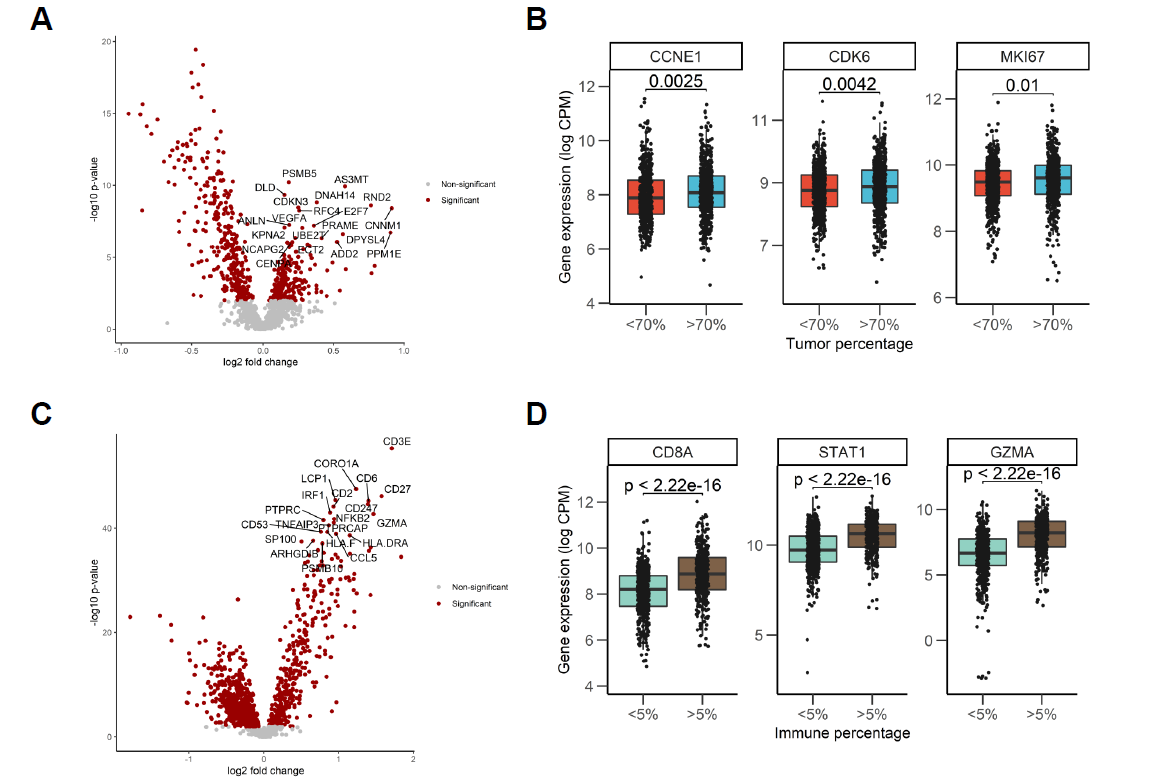


**Supplemental Figure S6. Differentially expressed genes between samples grouped by H&E staining. (A)** Scatterplot showing the log-transformed fold change (x-axis) and P-value (y-axis) of genes between samples with high (> 70%) and low (< 70%) tumor percentage. **(B)** Boxplot showing the expression level of representative tumor proliferation genes. **(C)** Scatterplot showing the log-transformed fold change (x-axis) and P-value (y-axis) of genes between samples with high (> 5%) and low (< 5%) immune percentage. **(D)** Boxplot showing the expression level of representative immune marker genes.


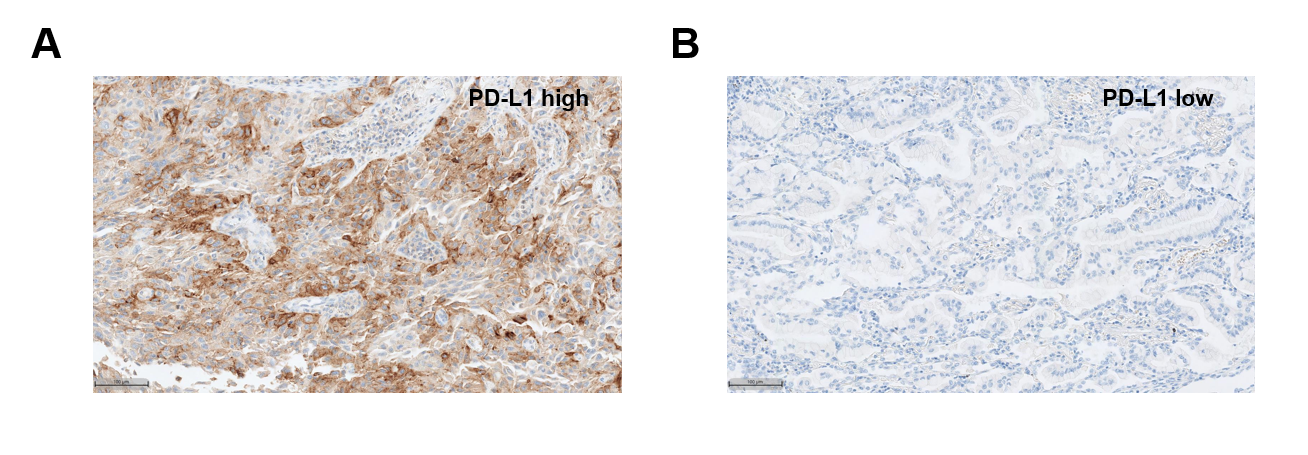


**Supplemental Figure S7. PD-L1 IHC staining on tumor cells. (A)** and **(B)** show the staining of PD-L1 (brown) on two representative lung tumor tissues. The scale bar is 100 μm.


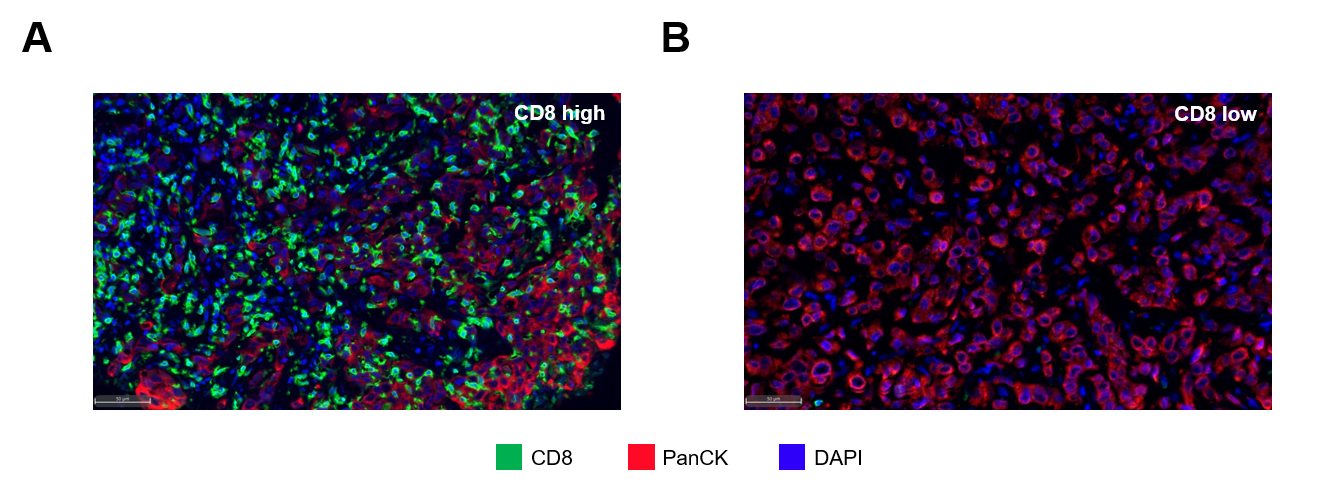


**Supplemental Figure S8. CD8 multiplex IHC staining on tumor area. (A)** and **(B)** show the staining of CD8 (green) and PanCK (red) on two representative tumor tissues in our study. The scale bar is 50 μm.
